# Successful Treatment of Complicated Pyogenic Spondylitis Due to Advanced Rectosigmoid Cancer Utilizing Vigorous Antibiotic Therapy and Minimally Invasive Robotic Colorectal Surgery: A Case Report

**DOI:** 10.7759/cureus.67536

**Published:** 2024-08-22

**Authors:** Keita Tanaka, Takahisa Fujikawa, Daibo Kojima, Keiji Nagata, Suguru Hasegawa

**Affiliations:** 1 Surgery, Kokura Memorial Hospital, Kitakyushu, JPN; 2 Gastroenterological Surgery, Fukuoka University Hospital, Fukuoka, JPN

**Keywords:** minimally invasive surgery, robotic colorectal surgery, batson venous plexus, pyogenic spondylitis, colorectal cancer

## Abstract

We report a case of rectosigmoid cancer complicated by pyogenic spondylitis. The patient was a 71-year-old man who had anemia and back pain. Endoscopy revealed a rectosigmoid tumor, confirmed to be well-differentiated adenocarcinoma. Imaging indicated rectosigmoid cancer with pyogenic spondylitis at the L1 vertebra. We performed radical resection (robotic-assisted Hartmann's procedure) after controlling the inflammation caused by pyogenic spondylitis. Colon cancer complicated by pyogenic spondylitis is rare. Here, we describe the mechanisms of this infection and treatment strategies along with a review of the literature.

## Introduction

Colonic bacteria in healthy people cannot enter the colonic tissue because they are covered by mucous membranes that serve a defense function for the body [1]. However, as the tumor grows and the local bacterial flora colonizes the tumor surface due to weak peristalsis or constipation, bacteria may invade the recently formed blood vessels and result in a distant hematogenous infection. The majority of hematogenous infections are liver abscesses via the portal vein, whereas infections of the spine, which have no venous access to the portal system, are extremely rare [2,3].

The main treatment for pyogenic spondylitis is local rest and antibiotics; moreover, long-term administration is required, lasting approximately six weeks [4]. Drainage and spinal fusion surgery are required if the infection is not controlled, an abscess develops, bone destruction progresses, or paralysis occurs [5,6].

Here, we present a case of rectosigmoid cancer complicated by pyogenic spondylitis, which was successfully treated with vigorous antibiotic therapy and subsequent minimally invasive robotic colorectal surgery.

## Case presentation

**Figure 1:**
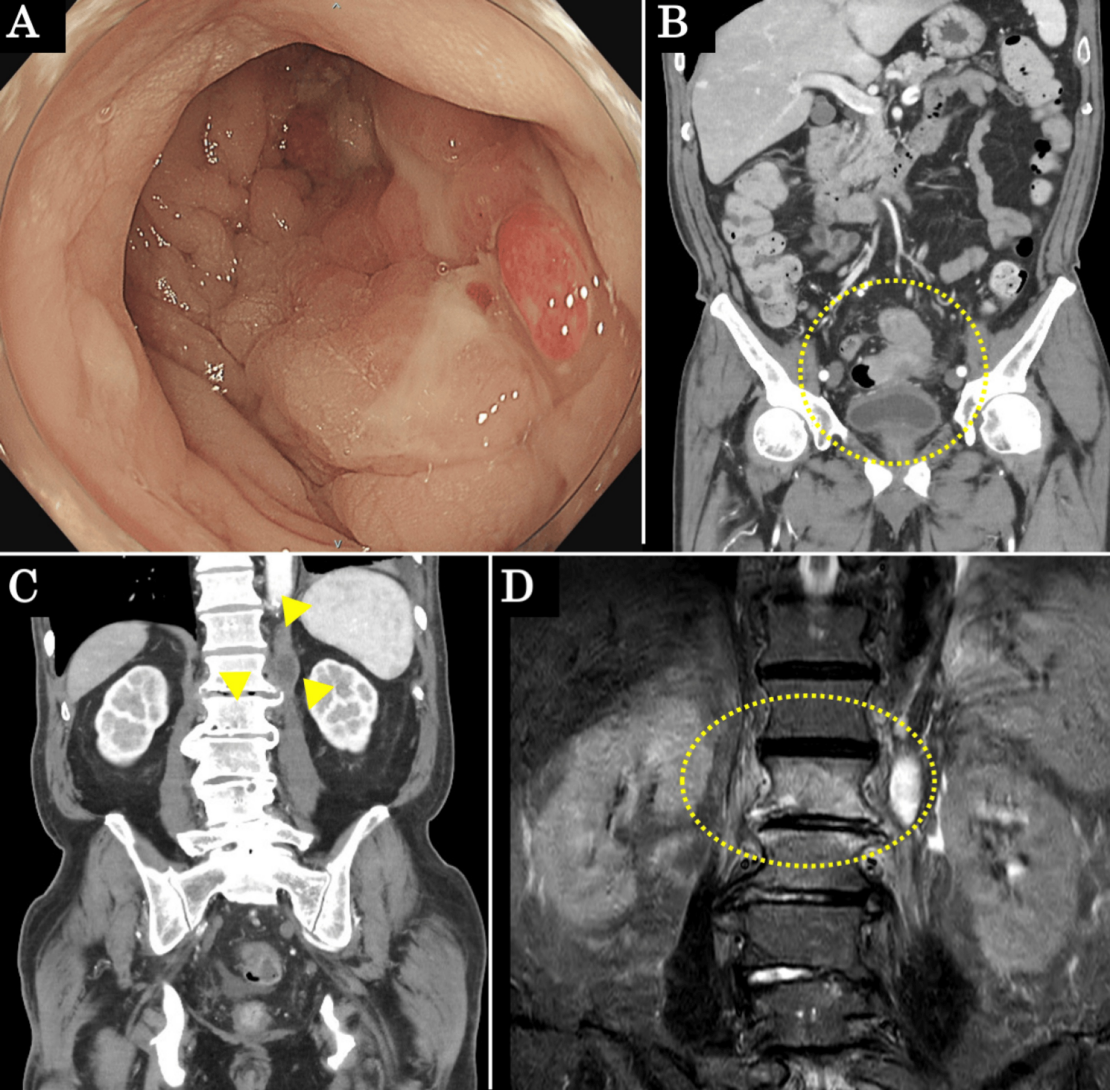
Preoperative endoscopy, contrast-enhanced CT, and MRI findings (A) Endoscopic findings revealed a circumferential tumor in the rectosigmoid. (B, C) Contrast-enhanced CT revealed wall thickening in the rectosigmoid, which was in contact with the small intestine and bladder (yellow dotted area). Fluid collection was observed on the left side of the L1 vertebral body (yellow arrows). (D) T2-weighted MRI revealed a high-intensity area on the L1 and L2 vertebral bodies, as well as the left side of the L1 vertebral body. (yellow dotted area).

A 71-year-old man presented to our hospital with complaints of anemia and lower back pain. On physical examination, his abdomen was soft with no tenderness, and no neurological findings were observed. The initial values of inflammatory markers were elevated as follows: white blood cell (WBC), 10800/μL (reference range: 3000-8900/μL); and C-reactive protein (CRP), 5.6 mg/dl (reference range: <0.5 mg/dl). The initial values of tumor markers were within normal limits, as follows: carcinoembryonic antigen (CEA), 1.0 ng/ml (reference range: <5 ng/ml); and carbohydrate antigen (CA19-9), 7 U/ml (reference range: <37 U/ml). Lower gastrointestinal endoscopy revealed a circumferential tumor that the scope found difficult to pass (Figure 1A), and a biopsy indicated well-differentiated adenocarcinoma. Contrast-enhanced computed tomography (CT) revealed wall thickening in rectosigmoid cancer, which was in contact with the small intestine and urinary bladder, as well as fluid collection on the left side of the L1 vertebral body (Figures 1B, 1C). T2-weighted magnetic resonance imaging (MRI) revealed a high-intensity area on the L1 and L2 vertebral bodies, as well as the left side of the L1 vertebral body (Figure 1D).

**Figure 2:**
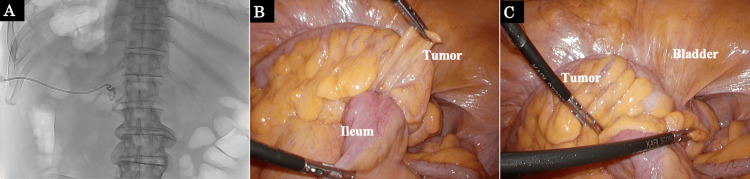
Abscess drainage findings and intraoperative findings during colostomy (A) Abscess drainage on the left side of the L1 vertebral body in the prone position. (B, C) The tumor was attached to the ileum and bladder.

The patient was diagnosed with rectosigmoid cancer (the eighth edition of the TNM (tumor classification: T4b, N1a, M0, Stage IIIC) and pyogenic spondylitis. First, abscess drainage was performed to control infection (Figure 2A), and the culture findings detected the presence of Eggerthella lenta. Because the bacteria are resident in the intestinal tract, spondylitis was diagnosed as a hematogenous infection caused by rectosigmoid cancer. Following abscess drainage, a laparoscopic colostomy was performed to prevent feces from passing through the cancer. Intraoperative findings showed that the tumor was attached to the ileum and bladder, indicating invasion (Figures 2B, 2C).

**Figure 3:**
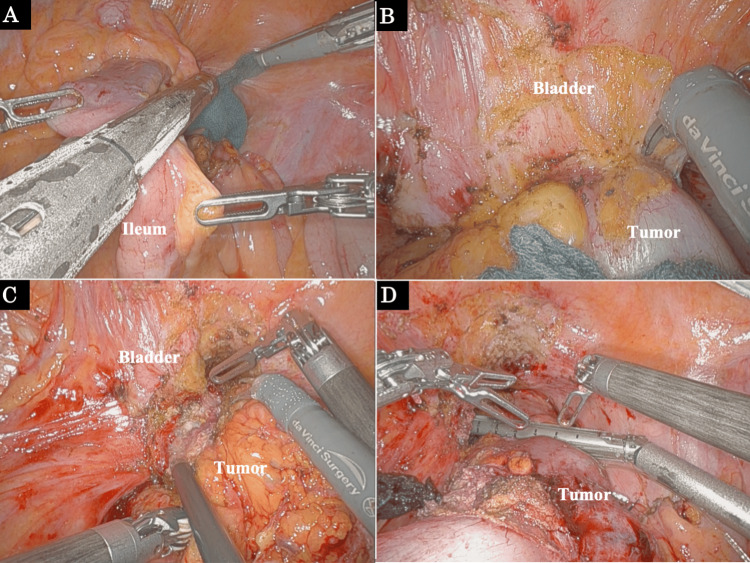
Operative findings during robot-assisted Hartmann’s procedure (A) The infiltrated part of the ileum was resected. (B, C) Findings around the bladder. There was no macroscopically obvious bladder invasion, and the bladder was preserved. (D) Rectal dissection.

After the inflammation improved with a colostomy and vigorous antibiotic therapy, we performed a radical cancer resection. We did not administer anticancer therapy prior to surgery to avoid a worsening of pyogenic spondylitis. First, we resected the ileum infiltrated with the tumor (Figure 3A). We suspected the tumor had invaded the bladder, but there was no obvious bladder invasion. A dissection between the tumor and the bladder was possible, and the bladder was preserved (Figures 3B, 3C). Although the splenic flexure was mobilized, it was hard to perform a colorectal anastomosis due to the inflamed, shortened mesocolon. We performed Hartmann’s procedure with central D3 lymph node dissection (Figure 3D).

**Figure 4:**
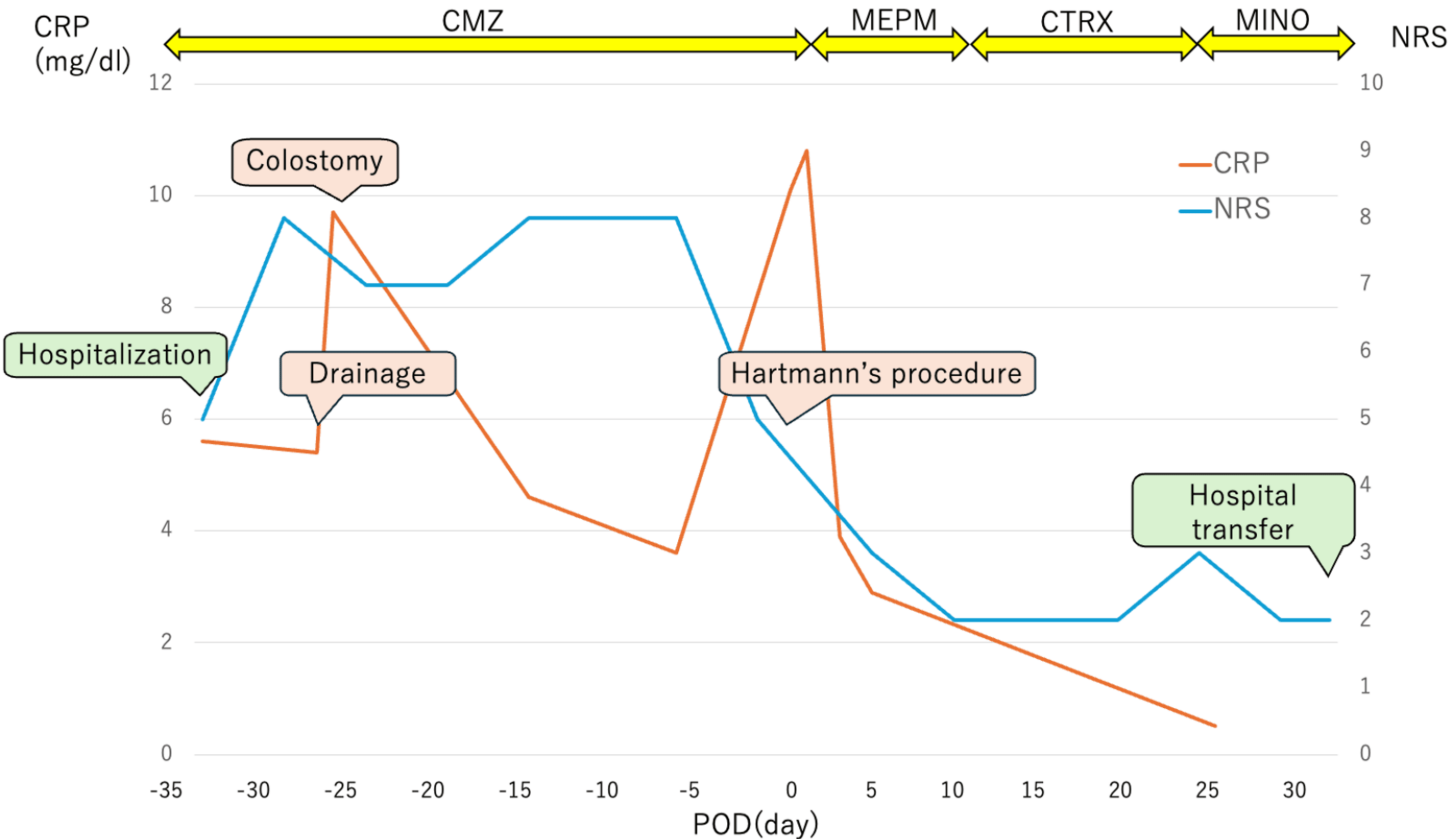
Clinical course of the current patient After abscess drainage, colostomy, and vigorous antibiotic therapy, Hartmann’s procedure was performed. Postoperatively, the patient's back pain dramatically improved, and the CRP level also improved. The patient was discharged on the thirty-third postoperative day without worsening back pain. CMZ: cefmetazole; MEPM: meropenem; CTRX: ceftriaxone; MINO: minocycline; CRP: C-reactive protein; NRS: numerical rating scale; POD: postoperative day

Figure 4 summarizes the perioperative clinical course of the current patient. Although lower back pain remained after abscess drainage, colostomy, and vigorous antibiotic therapy, lower back pain dramatically improved after tumor resection. There were no neurological symptoms in the lower extremities. We switched the antibiotics to oral administration after the CRP level had sufficiently improved. The patient's clinical course was uneventful, and the patient was transferred to the hospital for rehabilitation on the thirty-third postoperative day. Oral antibiotics were administered for a total of six months. The patient had an intravesical recurrence three months after the operation and began chemotherapy. The tumor was well controlled, chemotherapy has been continued without recurrence of pyogenic spondylitis, and the patient maintained good activities of daily living.

## Discussion

Colonic bacteria frequently cause hematogenous infection through colon cancer. Hematogenous infection typically causes liver abscesses, but infections in the spine, which lacks a portal vein system, are extremely rare. We assumed that bacteria from the colon caused a hematogenous infection through the Batson venous plexus, given the absence of any other sources of infection in this case. Batson established the Batson venous plexus in his study of the bone metastasis pathway of prostate cancer [7]. The rectum contains the rectal venous plexus, which joins with veins from other pelvic organs to form the pelvic venous plexus. The pelvic venous plexus connects to the Batson venous plexus, which consists of the internal vertebral venous plexus in the epidural space and the external vertebral venous plexus. The Batson venous plexus anatomically has thin walls and no valves, and it is thought that venous blood easily flows back from the pelvic venous plexus through the Batson venous plexus to the perivertebral body and epidural area [8]. Eggerthella lenta, which was confirmed by the drainage in the current case, is a resident bacteria in the intestinal tract. The rectosigmoid cancer was a circumferential lesion that was thought to have caused passage obstruction, resulting in increased intestinal pressure and pyogenic spondylitis via the Batson venous plexus.

Table 1 summarizes the reported cases of colorectal cancer complicated with pyogenic spondylitis. So far, 10 cases of coexisting colorectal cancer and pyogenic spondylitis have been reported including the current case [9-17]. There were seven cases in which spondylitis was diagnosed before colorectal cancer surgery, including this case. Except for one case in which the causative bacteria remained unidentified, all other reports indicated that the causative bacteria were resident colon bacteria. In all cases, the infection was treated before surgery, and surgery was performed after the inflammation was controlled.

**Table 1:** Reported cases of colorectal cancer complicated with pyogenic spondylitis post: postoperative; pre: preoperative; A/C: ascending colon; T/C: transverse colon; D/C: descending colon; S/C: sigmoid colon; MRSA: Methicillin-resistant *Staphylococcus aureus*

First Author	Year	Age	Sex	Diagnostic timing of spondylitis	Location of colorectal cancer	Causative bacteria
Katagiri et al. [9]	2002	56	female	post	S/C	Not described
Hiratsuka et al. [10]	2012	65	female	pre	RS-Ra	Klebsiella pneumonia
Masaki et al. [11]	2013	70	male	post	S/C	MRSA
Imai et al. [12]	2015	81	male	post	D/C	MRSA, Escherichia coli
Tajima et al. [13]	2015	70	male	pre	Ra	Klebsiella pneumoniae
Jaiswal et al. [14]	2017	63	male	pre	A/C	Klebsiella
Maruyama et al. [15]	2019	80	male	pre	D/C, S/C	Streptococcus bovis
Taguchi et al. [16]	2020	78	male	pre	T/C	Streptococcus gallolytics
Shibata et al. [17]	2022	72	male	pre	D/C	Pseudomonas aeruginosa
Current case	2024	71	male	pre	RS	Eggerthella lenta

In the present case, pyogenic spondylitis was diagnosed based on the presence of low back pain at the time of the diagnosis of rectosigmoid cancer. It has been reported that patients with unexplained abscess formation require gastrointestinal investigation [18]. Regarding the timing of surgery, it was performed after infection control as in previous reports. The postoperative course was favorable, and we believe that the timing of surgery was appropriate. During surgery, the head was positioned slightly lower and pressure was relieved as necessary to avoid excessive pressure that could lead to bone damage. 

Regarding surgery, we performed minimally invasive robotic surgery. Concerning robotic colorectal resection, it was initially documented by Weber et al. in 2002 [19] and has been reported frequently since then. Robotic surgery has been reported to be equivalent to open or laparoscopic surgery in terms of oncological, functional, and patient recovery outcomes [20]. Robotic surgery provides us with enlarged views of small structures, three-dimensional surgical views, reduced hand tremors, and instrument flexibility. We believe that these points have merit even in locally advanced colorectal cancer such as this case.

## Conclusions

We experienced a rare case of advanced rectosigmoid cancer complicated with pyogenic spondylitis. We prioritized infection control and were able to safely perform minimally invasive surgery without worsening the symptoms of pyogenic spondylitis. Although there is no established consensus on the treatment of colorectal cancer complicated by pyogenic spondylitis, it is considered desirable to control local inflammation and perform curative resection. This case highlights the importance of considering gastrointestinal sources in patients with pyogenic spondylitis of unknown origin.
